# Editorial: Statistical and Computational Methods for Microbiome Multi-Omics Data

**DOI:** 10.3389/fgene.2020.00927

**Published:** 2020-08-25

**Authors:** Himel Mallick, Vanni Bucci, Lingling An

**Affiliations:** ^1^Biostatistics and Research Decision Sciences, Merck & Co., Inc., Rahway, NJ, United States; ^2^Department of Microbiology and Physiological Systems, University of Massachusetts Medical School, Worcester, MA, United States; ^3^Interdisciplinary Program in Statistics and Data Science, The University of Arizona, Tucson, AZ, United States; ^4^Department of Epidemiology and Biostatistics, The University of Arizona, Tucson, AZ, United States; ^5^Department of Biosystems Engineering, The University of Arizona, Tucson, AZ, United States

**Keywords:** microbiome, metagenomics, metabolomics, multi-omics, biostatistics, computational biology, statistical genomics, data science

There has never been a more exciting time to do microbiome research thanks to the recent completion of several population-scale, longitudinal multi-omics studies including the NIH integrative human microbiome project (iHMP; iHMP Consortium, 2019) that have facilitated a multitude of new avenues of research for future investigations. These breakthroughs utilizing multiple ‘omics technologies have paved the way toward investigating biological systems at an unprecedented level of detail, allowing a simultaneous assessment of community function, dynamics, and biochemical signatures across diverse disease states and environments. The field of microbiome multi-omics, however, has not yet reached the maturity attained in other established molecular epidemiology fields such as cancer biomarker discovery and genome-wide association studies (Mallick et al., 2017). As a result, it remains wide open to an in-depth exploration of new analytical methods in order to make the leap from bench to bedside.

This Research Topic is a timely endeavor toward this goal to expand our knowledge on systems biology approaches in understanding microbial communities. Due to the complexity of the associated data, the downstream analysis of microbiome multi-omics remains challenging. While most of the initial studies focused on analyzing single omics (e.g., taxonomic or functional profiles), there has been a shift in the field toward the concurrent investigation of the microbiome and host phenotypes (e.g., metabolomics and host transcriptomics). To this end, many of the articles in this Research Topic focus on new ways to analyze and integrate multi-table data using cutting-edge statistical and computational methods.

Sankaran and Holmes revisit an overwhelmingly large literature and algorithms already available on multi-table data analysis by reviewing both the algorithmic foundations and practical applications of a wide range of analysis approaches and re-evaluate these paradigms with respect to heterogeneity, dimensionality, and sparsity in a fully reproducible setup. In a similar vein, Bodein et al. propose a computational framework to integrate longitudinal microbiome data with other omics and clinical data generated on the same biological specimens based on smoothing splines and multivariate dimension reduction methods. Both these constitute a critical contribution to the field, given the growing commonality of multi-table datasets and the complexity of related study designs, including dietary, pharmaceutical, clinical, and environmental covariates, often with samples from multiple time points or tissues.

Many important questions on microbiome multi-omics data integration remain unaddressed, especially those relating to extracting disease-relevant mechanistic networks that can provide insight into the complex web of host-microbiome interactions. Jiang et al. extensively review statistical aspects of relevant microbiome multi-omics network analysis methods by demystifying each class of methods with respect to their practical applicability and biological interpretability. Zhou and Gallins present a tutorial overview of commonly-used machine learning methods for microbiome host trait prediction, accompanied by validated R/Python implementations. The open-access source codes from these publications not only provide an important resource for algorithm developers but also ensure widespread usage and impact of these methods, facilitating future methodological research advances.

Moving beyond routine univariate analysis methods that ignore the correlations between features, Banerjee et al. take a multivariate approach to differential abundance analysis by jointly modeling all features in a set while maintaining the correct type I error and high power, which is not trivial for many existing per-feature methods (McMurdie and Holmes, 2014; Mandal et al., 2015; Jonsson et al., 2016, 2017; Thorsen et al., 2016; Mallick et al., 2017; Weiss et al., 2017; Hawinkel et al., 2019). Koh et al. introduce a distance-based kernel association test for family-based or longitudinal microbiome studies to associate microbial community composition with any type of host traits based on the generalized linear mixed model, vastly expanding the capability to incorporate non-Gaussian host traits as well as multiple kernels.

Quantitative methods of microbiome multi-omics are by no means limited to downstream analysis of targeted amplicon-based and metagenomic profiling. This Research Topic also contains papers addressing important questions in upstream data processing and quantitative microbiome profiling. For instance, Song et al. focus on the comparison of metagenomic samples using alignment-free methods with reads binning and conclude that alignment-free and alignment-based methods for metagenome comparison complement each other and should be used interactively to understand the dynamics of microbial communities. Yoon et al. estimate feature-feature correlations and partial correlations from robust measurements of microbial cell count, in particular, flow cytometry, and validate the results in a recent quantitative gut microbiome dataset ensuring both statistical rigor and biological relevance.

Several articles in the Research Topic go beyond integrating multiple omics datasets to establishing causation and molecular mechanism, with an emphasis on methods that aim to detect microbiome-mediated signals through causal mediation analysis. While existing methods in this space make strong parametric assumptions, which can be quite detrimental when the assumptions are violated, Carter et al. turn to nonparametric entropy models to detect significant mediation effects in the presence of high-dimensional exposures and mediators. Tang et al. utilize state-of-the-art microbiome compositional mediation analysis procedures to investigate the diet-microbiome-metabolome interaction in cross-sectional multi-omics samples from healthy subjects. Both these analyses estimate the total mediation effects of microbiome composition, as well as feature-specific mediation effects, providing additional mechanistic insights above and beyond a direct causal relationship.

Taken together, the papers in this Research Topic represent both an incredible amount of progress and an enormous potential for further advances in the near future. As a result, we have launched a second edition of the Research Topic where we will continue to add additional methods, research, and review articles over the next year or so.

## Author Contributions

All authors listed have made a substantial, direct and intellectual contribution to the work, and approved it for publication.

## Conflict of Interest

HM is employed by Merck Sharp & Dohme Corp., a subsidiary of Merck & Co., Inc., Kenilworth, NJ, USA. The remaining authors declare that the research was conducted in the absence of any commercial or financial relationships that could be construed as a potential conflict of interest.
